# Maternal supraphysiological hypercholesterolemia associates with endothelial dysfunction of the placental microvasculature

**DOI:** 10.1038/s41598-018-25985-6

**Published:** 2018-05-16

**Authors:** Bárbara Fuenzalida, Bastián Sobrevia, Claudette Cantin, Lorena Carvajal, Rocío Salsoso, Jaime Gutiérrez, Susana Contreras-Duarte, Luis Sobrevia, Andrea Leiva

**Affiliations:** 1 Cellular and Molecular Physiology Laboratory (CMPL), Division of Obstetrics and Gynaecology, School of Medicine, Faculty of Medicine, Pontificia Universidad Católica de Chile, Santiago, 8330024 Chile; 2 Cellular Signalling and Differentiation Laboratory (CSDL), School of Medical Technology, Health Sciences Faculty, Universidad San Sebastian, Santiago, 7510157 Chile; 3 Department of Physiology, Faculty of Pharmacy, Universidad de Sevilla, Seville, E-41012 Spain; 4 University of Queensland Centre for Clinical Research (UQCCR), Faculty of Medicine and Biomedical Sciences, University of Queensland, Herston, QLD 4029 Queensland Australia

## Abstract

Maternal physiological or supraphysiological hypercholesterolemia (MPH, MSPH) occurs during pregnancy. MSPH is associated with foetal endothelial dysfunction and atherosclerosis. However, the potential effects of MSPH on placental microvasculature are unknown. The aim of this study was to determine whether MSPH alters endothelial function in the placental microvasculature both *ex vivo* in venules and arterioles from the placental villi and *in vitro* in primary cultures of placental microvascular endothelial cells (hPMEC). Total cholesterol < 280 mg/dL indicated MPH, and total cholesterol ≥280 mg/dL indicated MSPH. The maximal relaxation to histamine, calcitonin gene-related peptide and adenosine was reduced in MSPH venule and arteriole rings. In hPMEC from MSPH placentas, nitric oxide synthase (NOS) activity and L-arginine transport were reduced without changes in arginase activity or the protein levels of endothelial NOS (eNOS), human cationic amino acid 1 (hCAT-1), hCAT-2A/B or arginase II compared with hPMEC from MPH placentas. In addition, it was shown that adenosine acts as a vasodilator of the placental microvasculature and that NOS is active in hPMEC. We conclude that MSPH alters placental microvascular endothelial function via a NOS/L-arginine imbalance. This work also reinforces the concept that placental endothelial cells from the macro- and microvasculature respond differentially to the same pathological condition.

## Introduction

Maternal levels of total cholesterol (TC) increase as human pregnancy progresses, i.e., maternal physiological hypercholesterolemia in pregnancy (MPH), to satisfy the demand of this lipid by the growing foetus^1^. However, some pregnant women show an excessive increase in TC, mainly due to an increase in the low-density lipoprotein cholesterol (LDL) level, a condition regarded as maternal supraphysiological hypercholesterolemia in pregnancy (MSPH)^2,3^. MSPH and increased maternal LDL levels are associated with endothelial dysfunction of the umbilical vein^1,3–5^ and early development of atherosclerosis in the foetal aorta^6,7^. MSPH is diagnosed in women with TC levels over a *cut-off* value of 280–290 mg/dL at term or over the 75th percentile for the different trimesters of pregnancy^4,6–10^. MSPH is associated with impaired nitric oxide (NO)-dependent dilation of human umbilical vein rings^3,4^. NO is synthesized by nitric oxide synthases (NOS). Particularly, endothelial NOS (eNOS) metabolize the amino acid L-arginine to nitric oxide and citrulline^1^. In placental endothelial cells, L-arginine is transported to the intracellular space by the human cationic amino acid transporter 1 (hCAT1), 2A and 2B (hCAT 2 A/B)^11,12^. In addition, L-arginine can be metabolized by other enzymes as arginases that compete with eNOS for substrate^13,14^. In human umbilical vein endothelial cells (HUVEC) isolated from MSPH pregnancies, reduced NOS activity, increased endothelial L-arginine uptake, and increased expression and activity of arginase II have been reported^3,5^. In addition, foetuses developed under MSPH conditions have increased atherosclerotic lesions in the aorta at birth^6^ and in childhood^7^. This phenomenon is also associated with increased markers of oxidative stress both in HUVEC from MSPH pregnancies^5^ and in maternal and neonatal circulation^8^. Therefore, it has been proposed that MSPH could be a crucial condition that increases the risk of an adverse foetal outcome and risk of developing cardiovascular diseases later in life^1,15,16^. In this scenario, the role of the placenta and the consequences of MSPH on the endothelial function of the placental microvasculature are still unknown.

Endothelial cells from different organs as well as from large and small vessels (i.e., macro- and microvasculature) of the same organ have specialized properties. In the human placenta, microvascular endothelial cells (hPMEC) function as a metabolic barrier, maintaining proper nutrient exchange between maternal and foetal circulation^11,17,18^. The expression of eNOS has been described in endothelial cells from the microvasculature; however, the activity or modulation of this enzyme by maternal pathological conditions such as MSPH is unknown^11,19^.

Therefore, the aim of this work was to determine the effects of MSPH on endothelial function in small vessels of the placenta *ex vivo* in microvenules and arterioles from the placental villi as well as *in vitro* in primary cultures of placental microvascular endothelial cells.

## Methods

### Study groups

Human placentas were collected from 20 normal full-term pregnancies (Table 1) from the Hospital Clínico UC-CHRISTUS (HCUC). The investigation conformed to the principles outlined in the Declaration of Helsinki. Ethics committee approval from the Faculty of Medicine of the Pontificia Universidad Católica de Chile (authorization number 11-066) and informed consent from patients were obtained. General maternal (i.e., age, height, weight, blood pressure and glucose levels) and neonatal (i.e., sex, gestational age, weight and height) variables were obtained from the clinical records. All pregnant women were screened for plasma levels of TC, high-density lipoprotein (HDL), LDL or very-low-density lipoprotein (VLDL) cholesterol and triglycerides (TG) at term of pregnancy (third trimester)^3,4^. Women with TC < 280 mg/dL were considered to have MPH, and those with TC ≥ 280 mg/dL were considered to have MSPH; these *cut-off* values for MSPH reflect values at which human fetoplacental endothelial and vascular dysfunction have been observed, as previously reported^3–8^. The exclusion criteria were maternal obesity at term of pregnancy, pre-gestational and gestational diabetes, preeclampsia, intrauterine growth restriction, foetal malformations or other maternal pathologies. Thus, among the 116 women who participated in this study, only 20 were included. The remaining 96 women were excluded based on the exclusion criteria.

**Table 1:** Clinical characteristics of pregnant women and new-borns.

Variables	MPH (*n* = 12)	MSPH (*n* = 8)
***Maternal variables***		
Age (years)	30.6 ± 1.4 (19–36)	30 ± 1.9 (25–39)
Height (cm)	161 ± 1.6 (152–168)	161 ± 1.9 (156–167)
Weight at delivery (kg)	74.4 ± 2.1 (61–85)	72 ± 3.8 (64–89)
BMI at delivery (kg/m^2^)	29.4 ± 1 (27–30)	28.5 ± 1.9 (23–30)
Mean arterial pressure at delivery (mmHg)	83.7 ± 1.8 (77–97)	82.8 ± 2.7 (70–91)
Basal glycemia (mg/dL)	76.7 ± 2 (68–84)	76.5 ± 3.5 (73–80)
OGTT (mg/dL)		
Basal glycemia	77.4 ± 1.6 (73–82)	76.5 ± 3.5 (73–80)
Glycemia 2 h after glucose	97.0 ± 4.4 (87–112)	99.5 ± 14.5 (85–114)
Lipid levels at delivery (mg/dL)		
Total cholesterol	234.6 ± 33 (183–275)	331 ± 16* (295–356)
HDL	64.4 ± 16 (48–100)	61 ± 12 (49–80)
LDL	117 ± 26 (80–150)	190.1 ± 39* (129–227)
VLDL	62.6 ± 28 (36–132)	58.4 ± 25 (49–68)
Triglycerides	246.9 ± 93 (80–426)	292 ± 50 (244–342)
***New-born variables***		
Sex (female/male)	7/5	4/4
Gestational age (weeks)	39.3 ± 0.4 (37–41)	39.1 ± 0.7 (37–41)
Birth weight (grams)	3536 ± 140 (2580–4170)	3490 ± 144 (2900–4000)
Height (cm)	51.2 ± 0.8 (45–54)	52.2 ± 0.8 (50–56)
Ponderal index (grams/cm^3^ × 100)	2.63 ± 0.08 (2.22–3.11)	2.45 ± 0.08 (2.07–2.76)

Women with maternal physiological (MPH, TC < 280 mg/dL) or supraphysiological hypercholesterolemia (MSPH, TC ≥ 280 mg/dL) at delivery were included (see Methods). Weight, body mass index (BMI), blood pressure and lipid profiles were determined at delivery. OGTT, oral glucose tolerance test. HDL, high-density lipoproteins; LDL, low-density lipoproteins; and VLDL, very-low-density lipoproteins. *P < 0.05 versus corresponding values in the MPH group. Data are presented as the mean ± S.D. (range).

### Plasma cholesterol and triglyceride measurement

TC, HDL, LDL, VLDL and TG levels were determined in maternal plasma obtained from brachial venous blood at the time of delivery. Lipid determination was performed in the Clinical Laboratory of the HCUC via standard enzymatic-colorimetric assays as previously described^3^.

### Human placental microvascular reactivity

Isometric force was measured in a myograph using microvessels isolated from placental type I stem villous^20–24^. In brief, isolated cotyledons were obtained from the placenta, and the chorionic plate and decidua were removed. Small vessels were identified under a stereomicroscope and dissected from the surrounding trophoblastic and connective tissue. Arterioles and venules (arterioles: 200–300 μm; venules: 200–500 μm) were identified by size and morphology and were carefully cut into rings of approximately 2 mm in length. The vessels were mounted onto a DMT 620 M wire myograph.

The internal diameter of the vessels as well as the optimal diameter for all the experiments was determined by the normalization module of the myograph (DMT normalization module in AD Instruments LabChart 7 software). All the rings were normalized to a luminal pressure of 50 mmHg^21^. The vessels were pre-constricted with 32.5 mmol/L KCl or 1 μmol/L serotonin, and then, the dilation in response to histamine (0.01–1000 μmol/L, 5 min)^21,25^, calcitonin gene-related peptide^23^ (CGRP, 0.01–1000 nmol/L, 5 min) or adenosine (0.01–1000 μmol/L, 5 min) was recorded in the absence or presence of 100 μmol/L *N*^G^-nitro-L-arginine methyl ester (L-NAME, NOS inhibitor, 30 min) or 10 μmol/L sodium nitroprusside (SNP, NO donor, 5 min), as previously reported^24,25^. Relaxation of the vessels was expressed as the percentage of relaxation caused by the different molecules compared to maximal contraction. The concentration of the drug required to produce 50 percent of the maximum relaxation (EC_50_) was calculated using the program GraphPad Prism.

### Cell culture

Human placental endothelial microvascular cells (hPMEC) were isolated by trypsin/collagenase digestion from placental villous tissue from MPH or MSPH pregnancies and cultured as described elsewhere^26–28^. In brief, placental sections were obtained after delivery, and the decidua and chorionic plate were removed. After that, larger vessels and calcifications were removed, and the tissue was carefully minced. The tissue was later digested with trypsin/EDTA (final concentration: 0.05%, 20 min, 37 °C). The digestion was stopped with foetal bovine serum (FBS, final concentration: 1%), and the tissue was centrifuged (1000 RCF, 5 min, 4 °C). The tissue was later digested with type II collagenase (0.1 mg/mL, 2 h, 37 °C) (Worthington, USA) in culture medium 199 (M199). After that, the digestion was stopped with M199 supplemented with FBS (final concentration : 1%) and centrifuged (300 RCF, 5 min, 4 °C). The supernatant, which contained the cells, was obtained and centrifuged (1500 RCF, 10 min, 4 °C). The cellular pellet was cultured in M199 supplemented with FBS (20%) and new-born calf serum (NBCS, 20%). After confluence, the cells were trypsinized and subjected to positive immunoselection using CD31 Dynabeads (Thermo Fisher scientific, USA). Cells were cultured in M199 medium containing FBS (10%), NBCS (10%), 3.2 mmol/L L-glutamine and 100 u/mL penicillin-streptomycin under standard conditions (37 °C, 5% CO_2_). Cells were positive for CD31 and von Willebrand factor (endothelial markers) and negative for alpha smooth muscle (muscle marker) expression^11^.

### Western blotting

Total proteins were separated by polyacrylamide gel electrophoresis, transferred to polyvinylidene difluoride membranes and later probed with primary polyclonal rabbit anti-total eNOS (1:200, 2 h, room temperature), polyclonal goat anti-hCAT-1 (1:200, 18 h, 4 °C), polyclonal goat anti-hCAT-2A/B (1:200, 2 h, room temperature), or polyclonal rabbit anti-ARGII (1:200, 18 h, 4 °C) (Santa Cruz Biotechnology, Santa Cruz, CA, USA) and monoclonal mouse anti-ß-actin (1:5000, 1 h, room temperature) (Sigma Aldrich, Germany) antibodies as previously described^3^. Membranes were rinsed and incubated (1 h, room temperature) with secondary horseradish peroxidase-conjugated rabbit anti-goat, goat anti-rabbit or anti-mouse antibodies (Santa Cruz Biotechnology). Proteins were detected by enhanced chemiluminescence and quantified by densitometry (Supplementary information).

### NOS activity

NOS activity was assayed by determining the intracellular content of L-citrulline by high-performance liquid chromatography (HPLC) as previously reported^5,29^. Briefly, confluent hPMEC were incubated (30 min, 37 °C) with HEPES buffer solution ((mmol/L) 50 HEPES pH 7.4, 100 NaCl, 5 KCl, 2.5 CaCl_2_, 1 MgCl_2_) supplemented with 100 μmol/L L-arginine in the absence or presence of 100 μmol/L L-NAME. After that, the cells were homogenized by sonication, and the proteins were quantified (BCA Protein Assay Kit, Thermo Fisher Scientific). Proteins were precipitated by the addition of HClO_4_ (1.5 mol/L) and K_2_CO_3_ (2 mol/L). After centrifugation (10000 RCF, 2 min, 4 °C), the amino acids in the supernatant were determined. L-citrulline content was estimated in samples derivatized with 14 mmol/L sodium borate and 4 mmol/L o-phtalaldehyde and injected in a C18 column (HiQsil, KYA Tech, Japan) coupled to an HPLC system (PU2089s, Jasco, Japan). The mobile phase was 0.1 mol/L NaH_2_PO_4_, pH 5.0 and 17% acetonitrile (0.7 mL/min flux rate). Fluorescence was monitored at excitation and emission wavelengths of 340 and 450 nm, respectively. The L-citrulline concentration was determined against a standard curve with the software ChromPass 1.7 (ChromPass Chromatography Data System, Jasco, Japan). All the values were corrected by the protein content in each sample. L-NAME-inhibited L-citrulline formation was estimated by the difference between values obtained in cells in the absence and in the presence of L-NAME.

### L-arginine uptake and transport

L-Arginine uptake (125 μmol/L L-arginine) and L-arginine transport (0–1000 μmol/L L-arginine) (3 μCi/mL L-[^3^H]arginine (Perkin-Elmer, USA), 1 min, 37 °C) were measured in hPMEC pre-incubated (overnight) in M199 containing NBCS (1%) and FBS (1%), as previously described^30^. The overall initial transport rates (i.e., the linear uptake for 1 min) were adjusted to the Michaelis-Menten hyperbola, and the maximal velocity (V_max_) and apparent Michaelis-Menten constant (K_m_) were calculated as previously described^3,30^. Maximal L-arginine transport capacity was estimated as *V*_max_/*K*_m_. The relative effect of MSPH versus MPH on the maximal L-arginine transport capacity (^*MSPH/MPH*^*F)* was estimated as [(*V*_max_/*K*_m_)_MSPH_/(*V*_max_/*K*_m_)_MPH_]^3^.

### Arginase activity

Arginase activity was estimated as total urea production from L-arginine. Enzymatic activity was measured in 70 μg of protein obtained after the lysis of hPMEC, as previously described^3,31^. In brief, 70 μg of protein was pre-incubated (10 min, 55 °C) with 100 mmol/L MnCl_2_ in 50 mmol/L Tris-HCl (pH 7.5) and then mixed with L-arginine (50 mmol/L, 60 min, 37 °C, pH 7.4). The reaction was stopped by the addition (400 μL) of an acid mixture (H_2_SO_4_:H_3_PO_4_:H_2_O = 1:3:7 v/v). Finally, the reactions were incubated (45 min, 100 °C) in 9% a-isonitrosopropiophenone solution for colorimetric determination of urea by absorbance at 540 nm. Each assay was performed in duplicate, and enzymatic activity was expressed as pmol urea/μg protein/min.

### Statistical analysis

Values for maternal and neonatal characteristics are presented as the mean ± S.D. For *ex vivo* and *in vitro* assays, the values are presented as the mean ± S.E.M., where *n* indicates the number of placentas (MPH = 5 and MSPH = 7) or cell cultures (MPH = 6 and MSPH = 6) used for the *ex vivo* and *in vitro* assays, respectively. Comparisons between two or more groups were performed by non-parametric (Mann-Whitney or Friedman test, respectively) tests. P < 0.05 was considered statistically significant.

### Data availability

The datasets generated during and/or analysed during the current study are available from the corresponding author on reasonable request.

## Results

### Study groups

All the evaluated maternal and neonatal general variables were similar between MSPH and MPH pregnancies (Table 1).

### Maternal lipid levels

The maternal plasma TC and LDL levels at term of pregnancy were higher in MSPH pregnancies than in MPH pregnancies. HDL, VLDL and TG levels were not altered by MSPH (Table 1).

### Human placental microvascular reactivity

**Figure 1:**
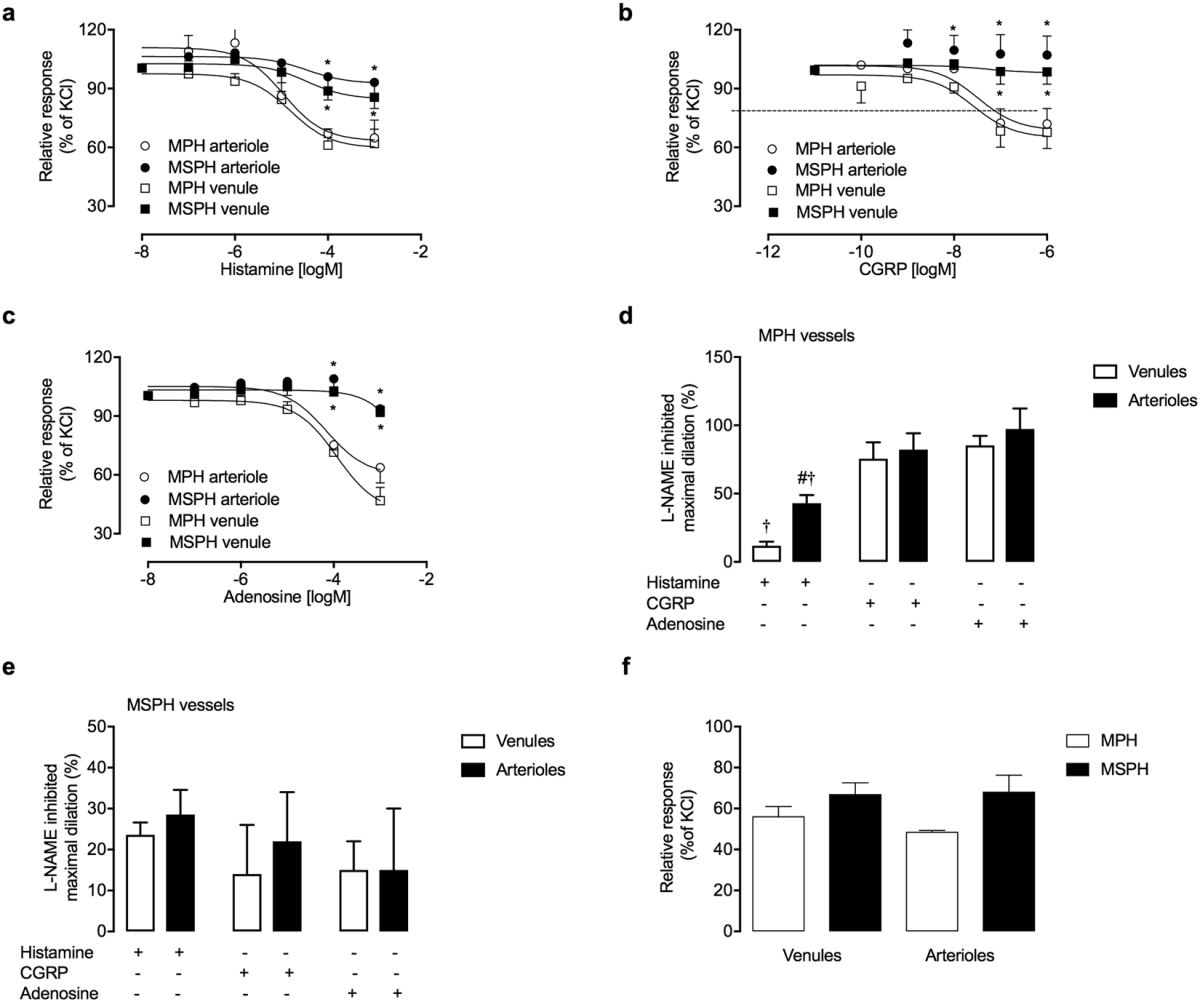
Human placental microvascular reactivity. Vascular dilation was determined in venule (□) and arteriole (○) rings obtained from human placental type I stem villous from pregnancies during which the mother exhibited MPH (white) or MSPH (black). Response to histamine (0.01–1000 μmol/L, 5 min, **a**), calcitonin gene-related peptide (0.01–1000 nmol/L, CGRP, 5 min, **b**) or adenosine (0.01–1000 μmol/L, 5 min, **c**) in vessels pre-incubated with KCl (32.5 mmol/L). The percentage of inhibition of maximal dilation in response to histamine, CGRP and adenosine was determined in the presence of *N*^G^-nitro-L-arginine methyl ester (L-NAME, 100 µmol/L, 30 min) in MPH (**d**) and MSPH (**e**) venules (white) and arterioles (black). (**f**) Dilation in response to sodium nitroprusside (10 μmol/L, 5 min) in vessels from MPH (white) or MSPH placentas (black). In (**a–c**) and (**f**), the data are expressed as a percentage of maximal dilation in response to KCl. *P < 0.05 versus the MPH values. ^#^*P* < 0.05 versus the venule values. ^**†**^*P* < 0.05 versus the CGRP and adenosine values. Values are presented as the mean ± S.E.M. (*n* = 5–7).

Endothelium-dependent vascular dilation was evaluated in venules and arterioles isolated from MPH and MSPH placentas. Histamine (Fig. 1a), CGRP (Fig. 1b) and adenosine (Fig. 1c) induced vascular dilation in venule and arteriole rings from the placental microvasculature (Table 2). Histamine, CGRP and adenosine caused lower maximal dilation (*D*_max_) in venule and arteriole rings from MSPH placentas than in those from MPH placentas (Fig. 1a–c, Table 2). In MPH vessels, inhibition of NOS activity with L-NAME caused a partial reduction in histamine-mediated dilation in venule (11.8 ± 3%) and arteriole (42.9 ± 6%) rings (Fig. 1d). However, CGRP- and adenosine-induced dilation was almost abolished in the presence of L-NAME both in venules and arterioles from MPH placentas (Fig. 1d). The effects of the inhibition of NOS activity on dilation (in the presence of L-NAME) were lower in MSPH venules and arterioles than in MPH vessels (Fig. 1e). To determine the effects of NO, the vessels were exposed to the NO donor SNP, which caused comparable dilation in rings from MPH and MSPH placentas (Fig. 1f). MSPH was not associated with changes in the *EC*_50_ for histamine in venule or arteriole rings or the *EC*_50_ for CGRP in venules (Table 2). The *EC*_50_ for CGRP in arterioles and the *EC*_50_ for adenosine in arterioles and venules from MSPH placentas were not measurable due to the reduced dilation of these vessels (Table 2).

**Table 2:** Dilation in human microvascular placental vessels.

Vasoactive molecule	*LogEC*_50_ (mol/L)		D_max_ (%)	
	MPH	MSPH	MPH	MSPH
**Histamine**				
Venule	−4.8 ± 0.27	−4.46 ± 0.39	38 ± 4.1	14.4 ± 6*
Arteriole	−4.91 ± 0.41	−4.45 ± 1.17	35.2 ± 5	6.9 ± 7.1*
**CGRP**				
Venule	−7.58 ± 0.44	−7.91 ± 3.24	32.3 ± 8.2	1.5 ± 6.3*
Arteriole	−7.48 ± 0.43	*n.m*	28 ± 7.8	−7 ± 9.7*
**Adenosine**				
Venule	−3.93 ± 0.17	*n.m*	53.2 ± 6.8	7.4 ± 2.7*
Arteriole	−4.16 ± 0.26	*n.m*.	36.3 ± 7.7	6.3 ± 4*

Placentas from women with maternal physiological (MPH, TC < 280 mg/dL) or supraphysiological hypercholesterolemia (MSPH, TC ≥ 280 mg/dL) in pregnancy were included (see Methods). CGRP, calcitonin gene-related peptide. Log EC_50_, half maximal effective concentration. D_max_, maximal dilation. *n.m*., not measurable. *P < 0.05 versus the corresponding values in the MPH group. Values are presented as the mean ± S.E.M (*n* = 3–7).

### NOS activity and eNOS expression

**Figure 2:**
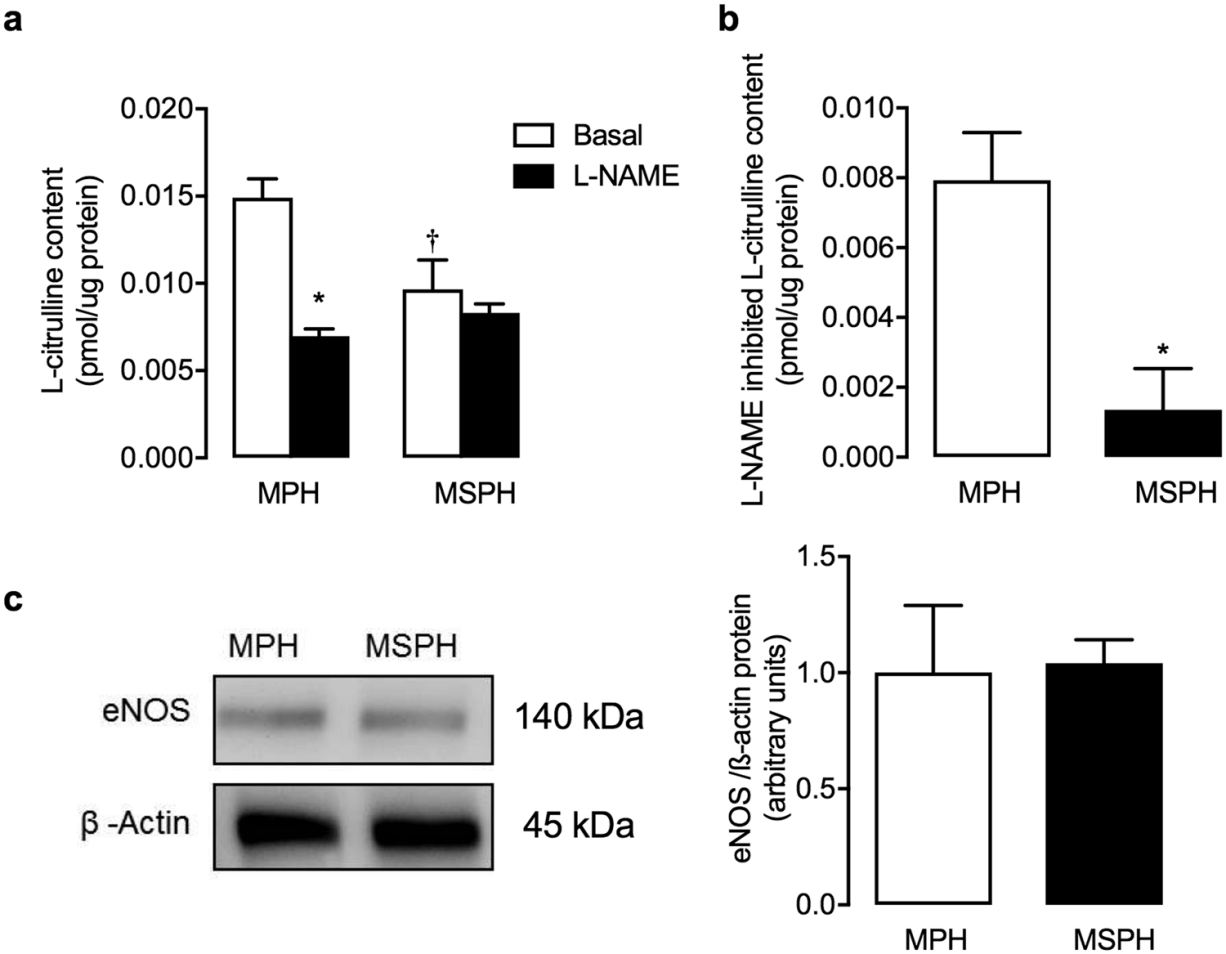
NOS activity. (**a**) L-citrulline content in hPMEC from pregnancies during which the mother exhibited MPH or MSPH. Cells were pre-incubated with L-arginine (100 μmol/L, 30 min) in the absence (white) or presence (black) of 100 μmol/L *N*^G^-nitro-L-arginine methyl ester (L-NAME, 30 min). (**b**) L-NAME inhibited L-citrulline content (from the data in a) in hPMEC from MPH (white) and MSPH (black) placentas. (**c**) Representative western blot for total eNOS expression in MPH or MSPH cells (ß-actin: internal control). *Lateral panel*: eNOS/ß-actin densitometry ratios normalized to 1 in the MPH group. In (**a**), *P < 0.05 versus values in the absence of L-NAME, ^†^*P* < 0.05 versus MPH values. In (**b**), *P < 0.05 versus MPH values. Values are presented as the mean ± S.E.M. (*n* = 6).

To determine NOS activity in endothelial cells from the microvasculature, L-citrulline formation was evaluated in hPMEC isolated from MPH and MSPH placentas. L-citrulline formation under basal conditions in hPMEC from MSPH placentas was lower than that in MPH cells, and incubation with L-NAME reduced L-citrulline formation in MPH cells but not in MSPH cells (Fig. 2a). Thus, L-NAME-inhibited L-citrulline formation was higher (5.87-fold) in MPH cells (0.00793 pmol/µg protein) than in MSPH cells (0.00135 pmol/µg protein) (Fig. 2b). Total eNOS protein abundance was not different between MSPH and MPH hPMEC (Fig. 2c).

### L-arginine transport

**Figure 3:**
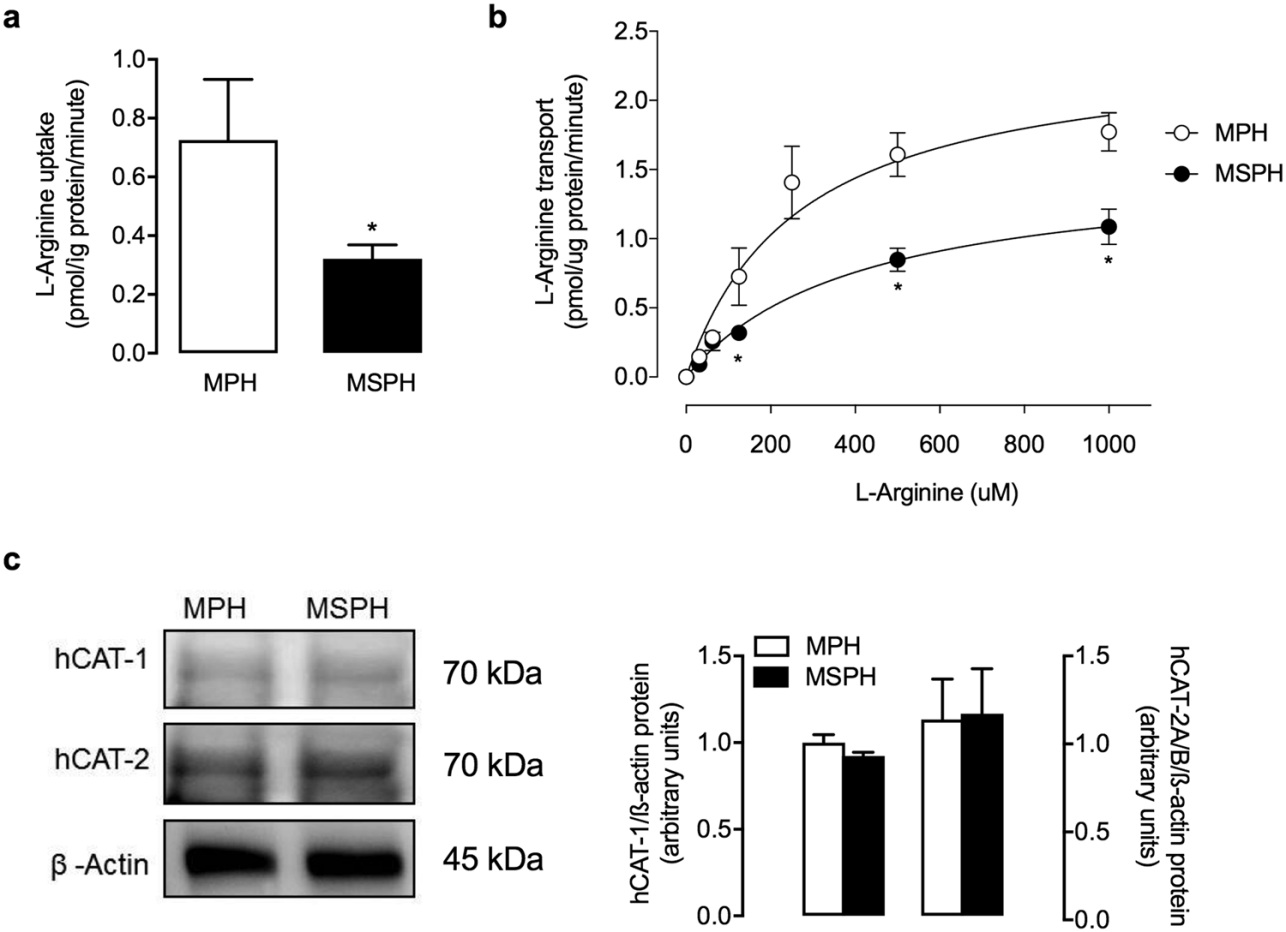
L-arginine transport. (**a**) Uptake of L-arginine (125 μmol/L, 1 min) in hPMEC from pregnancies during which the mother exhibited MPH (white) or MSPH (black). (**b**) Saturable L-arginine transport (0–1000 μmol/L, 1 min) in hPMEC from MPH or MSPH placentas. (**c**) Representative western blots for hCAT-1 and hCAT-2A/B in hPMEC from MPH or MSPH placentas (ß-actin: internal control). *Lateral panel*: hCAT-1 or hCAT-2A/B/ß-actin densitometry ratios normalized to 1 in the MPH group. *P < 0.05 versus MPH values. Values are presented as the mean ± S.E.M. (*n* = 6).

To determine the availability of the substrate of NOS for NO synthesis, the uptake of L-arginine was evaluated in hPMEC isolated from MPH and MSPH placentas. L-Arginine uptake at 125 μmol/L L-arginine was lower in hPMEC from MSPH placentas than in those from MPH placentas (Fig. 3a). To determine whether this change was also associated with changes in the kinetic parameters for the transport of the amino acid, the transport of L-arginine was evaluated. Overall L-arginine transport was saturable in MPH and MSPH cells (Fig. 3b). In hPMEC from MSPH placentas, L-arginine transport exhibited lower *V*_max_ and *V*_max_/*K*_m_ but higher *K*_m_ than that in cells from MPH placentas (Fig. 3b, Table 3). Thus, it was estimated that L-arginine transport, evaluated as the relative effect of MSPH versus MPH on the maximal L-arginine transport capacity (^*MSPH/MPH*^*F*), was reduced in cells from MSPH pregnancies (Table 3). Parallel assays showed that neither the protein abundance of hCAT-1 nor that of hCAT-2A/B was altered in cells from MSPH pregnancies compared with those from MPH pregnancies (Fig. 3c).

**Table 3:** Kinetic parameters for L-arginine transport.

Parameter	MPH	MSPH
*V*_max_ (pmol/µg protein/min)	2.39 ± 0.3	1.53 ± 0.18*
*K*_m_ (µmol/L)	266.2 ± 36.6	408.3 ± 48.6*
*V*_max_/*K*_m_ (pmol/µg protein/min/(µmol/L))	0.009 ± 0.001	0.004 ± 0.0004
^*MSPH/MPH*^ *F*	0.44 ± 0.04	

L-arginine transport was assayed (see Methods) in hPMEC isolated from placentas from pregnancies with maternal physiological (MPH, TC < 280 mg/dL) or supraphysiological hypercholesterolemia (MSPH, TC ≥ 280 mg/dL) (see Methods). *V*_max_, maximal velocity; and *K*_m_, apparent Michaelis-Menten constant. ^*MSPH/MPH*^*F* is the relative effect of MSPH versus MPH on the maximal L-arginine transport capacity (*V*_max_/*K*_m_). *P < 0.05 versus the corresponding values in the MPH group. Values are presented as the mean ± S.E.M. (*n* = 6).

### Arginase activity

**Figure 4:**
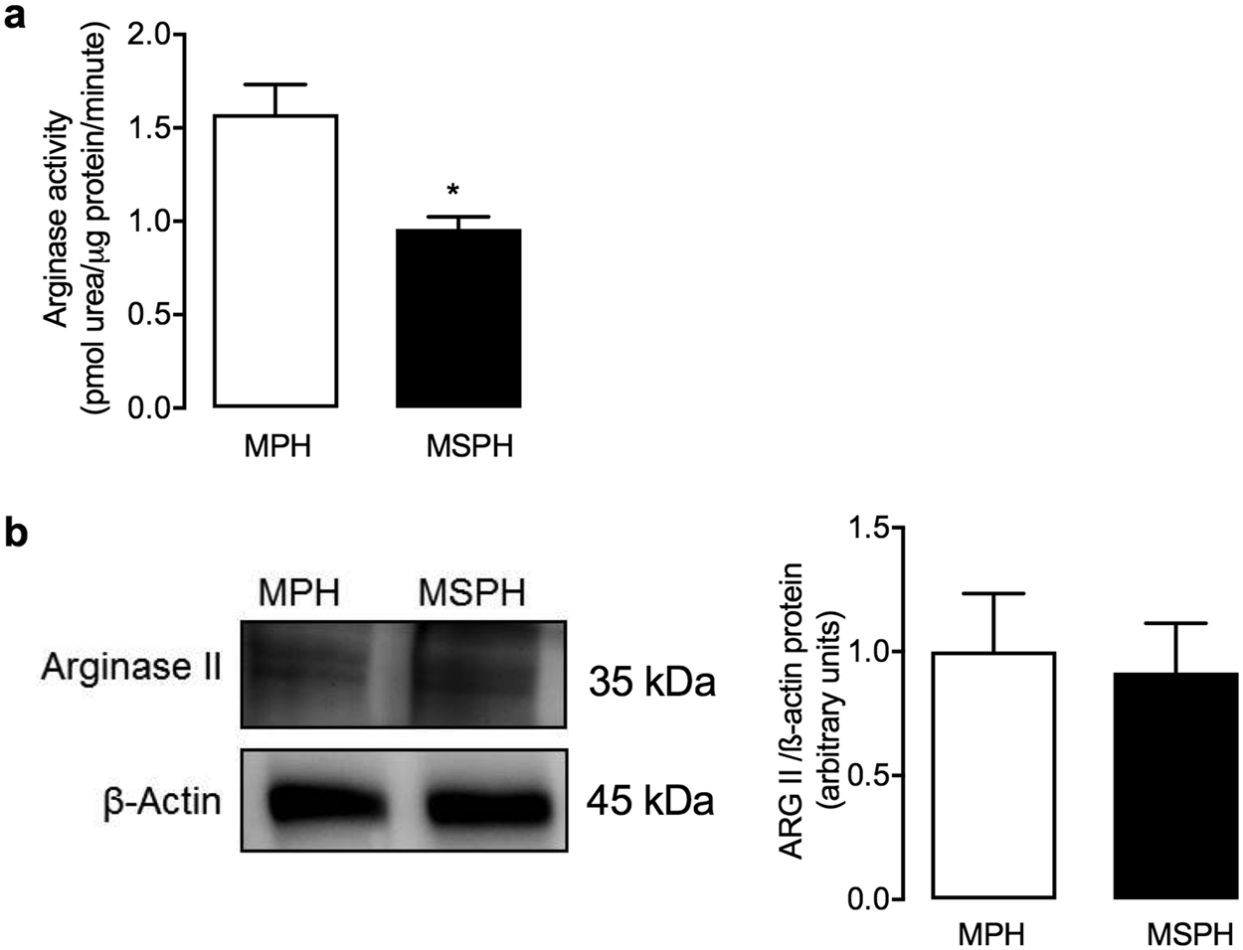
Arginase activity. (**a**) Arginase activity was evaluated as urea formation from L-arginine (50 mmol/L, 60 min, 37 °C) in hPMEC from pregnancies during which the mother exhibited MPH (white) or MSPH (black). (**b**) Representative western blot for arginase II in hPMEC from MPH or MSPH placentas (ß-actin: internal control). *Lateral panel*: Arginase II/ß-actin densitometry ratios normalized to 1 in the MPH group. *P < 0.05 versus MPH values. Values are presented as the mean ± S.E.M. (*n* = 6).

To determine a possible role for arginases in the reduced formation of L-citrulline in hPMEC from MSPH placentas, arginase activity was assayed in hPMEC isolated from MPH and MSPH placentas. Cells from MSPH placentas exhibited lower arginase activity than those from MPH placentas (Fig. 4a). Arginase I protein was not detectable (data not shown) in hPMEC, and arginase II protein abundance was not different between MSPH and MPH hPMEC (Fig. 4b).

## Discussion

This study shows for first time that increased levels of maternal TC and LDL in MSPH are associated with endothelial dysfunction of the human placental microvasculature, likely due to an imbalance between NOS activity and L-arginine transport.

We showed vascular alterations in the microvasculature of placentas from women with levels of TC over a *cut-off* value of 280 mg/dL. However, this value is suggested as a surrogate marker for placental dysfunction. At TC levels over 280 mg/dL, changes in both the foetal vasculature (i.e., increased foetal atherosclerosis^6,7^, markers of oxidative stress^8^ and differential fatty acid composition^8^ in the foetal blood) and the placenta (i.e., umbilical vein endothelial dysfunction^3–5^, changes in the placental expression of lipoprotein receptors and cholesterol transporters^9,10^) without changes in new-born size and weight, or in neonatal levels of TC, LDL, HDL, or TG have been described^3,4,9^. Despite that, determination of early markers of endothelial dysfunction or metabolic changes in the neonates from MSPH would be necessary to associate this maternal condition with changes in the neonatal outcome. In addition, increased maternal TC has also been associated with higher atherosclerotic lesions in the childhood^9^ and elevated lipid levels in the adulthood^32^ of the offspring. Thus, it has been proposed that MSPH may be a maternal condition required for proper placental function with consequences on foetal vascular health during pregnancy and later in life^1,16,32,33^.

There is cumulative experimental evidence showing that macrovascular and microvascular endothelial cell function is different, even within the same vascular bed; this phenomenon is required for proper vascular homeostasis^18,26,34^. Therefore, in addition to describing the detrimental effects of MSPH on endothelial function in the placental microvasculature, our study also shows that endothelial cells from the macro- and microvasculature respond to the same pathological conditions by reducing NOS activity; however, the mechanism leading to this reduction may be different.

Since the human placenta lacks innervation, local synthesis and release of vasoactive molecules lead to acute and rapid modulation of the vessel tone in this vascular bed^35^. In addition, classical endothelium-dependent vasodilators, such as acetylcholine or bradykinin, have minimal vasodilator effects on this vascular bed, and molecules such as histamine and CGRP have been used as vasodilators for fetoplacental vessels^21,23,25^. In this study, we showed for the first time that the nucleoside adenosine has vasodilator effects on the placental microvasculature both in venules and arterioles isolated from placental type I stem villous (Fig. 1c). As previously demonstrated in umbilical vein rings^36^, the vasodilator effect of adenosine on the placental microvasculature may depend on NOS activity in the endothelium, as was shown by the inhibition of its vasodilator effect in the presence of L-NAME (Fig. 2d,e). The effects of adenosine on this vascular bed may be due to and modulated by adenosine receptors (ARs) (i.e., A_2A_AR and A_2B_AR) and adenosine transporters (hENT1 and hENT2), which are functionally expressed in these microvascular endothelial cells^26,37^.

Regarding the effects of MSPH on microvascular dilation, we showed that the maximal response (D_*max*_) to histamine, CGRP and adenosine was reduced in venule and arteriole rings from MSPH placentas (Fig. 1a–c, Table 2) without changes in their sensitivity (EC_50_) (Table 2). Since the effects of CGRP and adenosine were almost abolished by NOS inhibition with L-NAME (Fig. 1d,e), we suggest that the response to these molecules is dependent on NOS activity in the endothelium, a phenomenon that may be impaired in MSPH vessels. In MSPH vessels, the inhibitory effect by L-NAME was reduced compared with that in MPH vessels, possibly because vessel relaxation was significantly lower in MSPH vessels than in MPH vessels (Fig. 1d,e). In addition, the effect of histamine on MPH vessels was partially inhibited by L-NAME, a result that is consistent with previous data showing that the effect of histamine on the placental vasculature depends on the expression of histamine receptors both in the endothelium and in the smooth muscle^38^. Since dilation caused by SNP (NO donor) was unaltered in MSPH vessels (Fig. 1f) and the L-NAME-inhibited response to CGRP and adenosine was reduced in MSPH vessels, we suggest that the endothelium, rather than the smooth muscle, is likely affected by MSPH. Thus, impaired microvascular endothelial function may be present in MSPH. However, these results need to be confirmed using endothelium-denuded vessels.

To determine whether placental microvascular endothelial cell function was altered in MSPH, NOS activity was evaluated in hPMEC isolated from MSPH placentas. Previous studies have shown that eNOS expression in hPMEC is lower than that in HUVEC or placental homogenate^11^ and that eNOS is expressed in the endothelium from the sub-chorionic plate villi but only slightly expressed in small vessels from the terminal villi^19,39,40^. However, NOS activity, as assayed by nitrite determination, was not detectable in hPMEC^11^. The placental tissue used for the preparation of hPMEC is heterogeneous; thus, isolation of microvascular endothelial cells from different areas of the placenta is expected, including microvascular vessels from the sub-chorionic plate, which express eNOS and produce NO. Our present study showed that NOS activity, evaluated as L-NAME-inhibited L-citrulline content, was measurable in hPMEC. Compared with MPH cells, NOS activity was lower in MSPH cells (Fig. 2b). Since in the placenta the main source of NO is eNOS, we suggest that NOS activity determined in this study relates with eNOS activity. However, the participation of the inducible NOS isoform (iNOS) needs to be determined. Since eNOS protein abundance was not different between MSPH and MPH (Fig. 2c), the lower CGRP- and adenosine-induced dilation in MSPH cells may have resulted from reduced NOS activity. These results were comparable to those described in HUVEC from MSPH pregnancies^3^.

Since L-arginine uptake is required for eNOS activity in endothelial cells^41,42^, reduced L-arginine uptake and further restricted delivery to eNOS may occur in cells from MSPH placentas. Our results show that hPMEC from MSPH placentas exhibit lower *V*_max_ and *V*_max_/*K*_m_ for L-arginine transport as well as higher *K*_m_ than those from MPH placentas (Fig. 3a,b, Table 3). Thus, eNOS should be metabolically less efficient in hPMEC from MSPH placentas than in those from MPH placentas due to lower L-arginine availability. Interestingly, this impaired L-arginine transport was not associated with changes in the total protein level of the L-arginine transporter hCAT-1 or hCAT-2A/B (Fig. 3c). This result is contrary to that described in HUVEC from MSPH pregnancies, in which L-arginine uptake was increased. Thus, this result reinforces the concept that the same condition has different effects on endothelial cells from the macro- or microvasculature.

Arginases metabolize L-arginine to urea in endothelial cells from the macro-^29,43^ and microvasculature^44,45^, thus competing with eNOS for substrates. Our results show that arginase activity was lower in hPMEC from MSPH placentas without changes in arginase II protein abundance (Fig. 4a,b). Arginase I was undetectable in hPMEC. This result differs from the results previously described in HUVEC from MSPH pregnancies, in which an increase in arginase activity was shown^3^. Thus, we suggest that arginases are not involved in the reduction of NOS activity described in hPMEC from MSPH placentas.

As shown, the reduced NOS activity and L-arginine uptake was not associated with changes in eNOS, hCAT-1 or hCAT-2A/B protein abundance. Cholesterol is a key constituent of lipid rafts and caveolae^46^. Thus, in conditions such as hypercholesterolemia, caveolin is up-regulated, leading to modifications of its localization and modifications in the signalling of proteins located in caveolae^47–49^, such as eNOS and hCATs, which are required for proper function and NO synthesis^50–52^. Thus, we speculate that in hPMEC from MSPH placentas, decreased NO synthesis may be related to alterations in hCAT and eNOS distribution in the plasma membrane microdomains without changes in protein abundance. However, this hypothesis needs to be evaluated in our model.

In human and animal models, a strong association of LDL and HDL levels with endothelial dysfunction and atherosclerosis has been established^53–55^. However, a possible pathogenic role for dyslipidaemia during pregnancy has only been suggested^3–7,16^. Maternal cholesterol carried in LDL and HDL is transferred across the trophoblast cells to the basal membrane and then to placental endothelium and neonatal lipoproteins by LDL and HDL receptors^56–59^ and cholesterol transporters such as the ATP binding cassette transporter sub-family A member 1 (ABCA1) and G member 1 (ABCG1)^59^. However, how maternal-derived cholesterol is transferred across the human placenta is still not fully understood. Expression of some of these receptors and transporters are deregulated in placentas from pregnancies with increased levels of TC and TG (mRNA and protein)^9,10^, suggesting that in MSPH, the placenta could modulate the uptake and delivery of maternal lipids. However, it is necessary to evaluate the possible pathological consequences of the increase in maternal cholesterol on cholesterol trafficking in the placenta (uptake and efflux) to confirm the implication of maternal dyslipidaemia in the placental vascular alterations described in this study. In addition, it has been reported that HDL modulates eNOS activity in the endothelium^49,55,60^. However, whether neonatal HDL from MSPH pregnancies also modulates eNOS activity in placental endothelium is unknown.

In summary, although MSPH treatment or prevention studies are necessary to establish a causal effect, we describe here, for the first time, that MSPH is a pathological maternal condition that impairs placental microvasculature dilation as well as NOS activity and L-arginine uptake in hPMEC without changes in arginase activity. Finally, the consequences of this maternal condition on the neonatal outcome needs to be determined.

## Electronic supplementary material

Supplementary information

## References

[1] Leiva A (2016). Nitric oxide is a central common metabolite in vascular dysfunction associated with diseases of human pregnancy. Curr. Vasc. Pharmacol..

[2] Montes A (1984). Physiologic and supraphysiologic increases in lipoprotein lipids and apoproteins in late pregnancy and postpartum. Possible markers for the diagnosis of ‘prelipemia’. Arteriosclerosis.

[3] Leiva A (2013). Maternal hypercholesterolemia in pregnancy associates with umbilical vein endothelial dysfunction: role of endothelial nitric oxide synthase and arginase II. Arterioscler. Thromb. Vasc. Biol..

[4] Leiva A (2015). Cross-sectional and longitudinal lipid determination studies in pregnant women reveal an association between increased maternal LDL cholesterol concentrations and reduced human umbilical vein relaxation. Placenta.

[5] Leiva A (2016). Tetrahydrobiopterin role in human umbilical vein endothelial dysfunction in maternal supraphysiological hypercholesterolemia. Biochim. Biophys. Acta.

[6] Napoli C (1997). Fatty streak formation occurs in human fetal aortas and is greatly enhanced by maternal hypercholesterolemia. Intimal accumulation of low density lipoprotein and its oxidation precede monocyte recruitment into early atherosclerotic lesions. J. Clin. Invest..

[7] Napoli C (1999). Influence of maternal hypercholesterolaemia during pregnancy on progression of early atherosclerotic lesions in childhood: Fate of Early Lesions in Children (FELIC) study. Lancet (London, England).

[8] Liguori A (2007). Effect of gestational hypercholesterolaemia on omental vasoreactivity, placental enzyme activity and transplacental passage of normal and oxidised fatty acids. BJOG.

[9] Ethier-Chiasson M (2007). Influence of maternal lipid profile on placental protein expression of LDLr and SR-BI. Biochem. Biophys. Res. Commun..

[10] Zhang R (2017). Modulation of cholesterol transport by maternal hypercholesterolemia in human full-term placenta. PLoS One.

[11] Dye JF (2004). Characterization of cationic amino acid transporters and expression of endothelial nitric oxide synthase in human placental microvascular endothelial cells. FASEB J..

[12] Sobrevia L (2016). Insulin Is a Key Modulator of Fetoplacental Endothelium Metabolic Disturbances in Gestational Diabetes Mellitus. Front Physiol..

[13] Ryoo S (2008). Endothelial arginase II: a novel target for the treatment of atherosclerosis. Circ Res..

[14] Pernow J, Jung C (2016). The Emerging Role of Arginase in Endothelial Dysfunction in Diabetes. Curr Vasc Pharmacol..

[15] Palinski W (2009). Maternal-fetal cholesterol transport in the placenta: good, bad, and target for modulation. Circ. Res..

[16] Palinski W (2014). Effect of maternal cardiovascular conditions and risk factors on offspring cardiovascular disease. Circulation.

[17] Leach L (2011). Placental vascular dysfunction in diabetic pregnancies: intimations of fetal cardiovascular disease?. Microcirculation.

[18] Sobrevia L (2011). Review: Differential placental macrovascular and microvascular endothelial dysfunction in gestational diabetes. Placenta.

[19] Myatt L, Brockman DE, Eis AL, Pollock JS (1993). Immunohistochemical localization of nitric oxide synthase in the human placenta. Placenta.

[20] Demir R, Kosanke G, Kohnen G, Kertschanska S, Kaufmann P (1997). Classification of human placental stem villi: review of structural and functional aspects. Microsc. Res. Tech..

[21] Wareing M, Crocker IP, Warren AY, Taggart MJ, Baker PN (2002). Characterization of small arteries isolated from the human placental chorionic plate. Placenta.

[22] Jerat S, Morrish DW, Davidge ST, Kaufman S (2001). Effect of adrenomedullin on placental arteries in normal and preeclamptic pregnancies. Hypertension.

[23] Dong YL (2004). Involvement of calcitonin gene-related peptide in control of human fetoplacental vascular tone. Am. J. Physiol. Heart Circ. Physiol..

[24] Ong SS, Crocker IP, Warren AY, Baker PN (2002). Functional characteristics of chorionic plate placental arteries from normal pregnant women and women with pre-eclampsia. Hypertens. pregnancy.

[25] Sabry S, Mondon F, Ferre F, Dinh-Xuan AT (1995). *In vitro* contractile and relaxant responses of human resistance placental stem villi arteries of healthy parturients: role of endothelium. Fundam. Clin. Pharmacol..

[26] Dye JF, Leach L, Clark P, Firth JA (2001). Cyclic AMP and acidic fibroblast growth factor have opposing effects on tight and adherens junctions in microvascular endothelial cells *in vitro*. Microvasc. Res..

[27] Lang I (2003). Heterogeneity of microvascular endothelial cells isolated from human term placenta and macrovascular umbilical vein endothelial cells. Eur. J. Cell Biol..

[28] Salomon C (2012). Gestational diabetes reduces adenosine transport in human placental microvascular endothelium, an effect reversed by insulin. PLoS One.

[29] Pardo F (2015). Human supraphysiological gestational weight gain and fetoplacental vascular dysfunction. Int. J. Obes. (Lond).

[30] Subiabre M (2017). Maternal insulin therapy does not restore foetoplacental endothelial dysfunction in gestational diabetes mellitus. Biochim. Biophys. Acta.

[31] Prieto CP (2011). Hypoxia-reduced nitric oxide synthase activity is partially explained by higher arginase-2 activity and cellular redistribution in human umbilical vein endothelium. Placenta.

[32] Mendelson MM (2016). Association of maternal prepregnancy dyslipidemia with adult offspring dyslipidemia in excess of anthropometric, lifestyle, and genetic factors in the Framingham heart study. JAMA Cardiol..

[33] Wild R, Weedin EA, Wilson D (2015). Dyslipidemia in pregnancy. Cardiol. Clin..

[34] Shi Y, Vanhoutte PM (2017). Macro- and microvascular endothelial dysfunction in diabetes. J. Diabetes.

[35] Marzioni D (2004). Restricted innervation of uterus and placenta during pregnancy: evidence for a role of the repelling signal Semaphorin 3A. Dev. Dyn..

[36] Westermeier F (2011). Insulin restores gestational diabetes mellitus-reduced adenosine transport involving differential expression of insulin receptor isoforms in human umbilical vein endothelium. Diabetes.

[37] Escudero C, Casanello P, Sobrevia L (2008). Human equilibrative nucleoside transporters 1 and 2 may be differentially modulated by A2B adenosine receptors in placenta microvascular endothelial cells from pre-eclampsia. Placenta.

[38] Mills TA, Taggart MJ, Greenwood SL, Baker PN, Wareing M (2007). Histamine-induced contraction and relaxation of placental chorionic plate arteries. Placenta.

[39] Buttery LD (1994). Endothelial nitric oxide synthase in the human placenta: regional distribution and proposed regulatory role at the feto-maternal interface. Placenta.

[40] Ghabour MS, Eis AL, Brockman DE, Pollock JS, Myatt L (1995). Immunohistochemical characterization of placental nitric oxide synthase expression in preeclampsia. Am. J. Obstet. Gynecol..

[41] Acevedo CG, Carrasco G, Burotto M, Rojas S, Bravo I (2001). Ethanol inhibits L-arginine uptake and enhances NO formation in human placenta. Life Sci..

[42] Xiao D, Bird IM, Magness RR, Longo LD, Zhang L (2001). Upregulation of eNOS in pregnant ovine uterine arteries by chronic hypoxia. Am. J. Physiol. Heart Circ. Physiol..

[43] Pandey, D. *et al*. OxLDL triggers retrograde translocation of arginase2 in aortic endothelial cells via ROCK and mitochondrial processing peptidase. *Circ. Res*. **115** 450–459 (2014). 10.1161/CIRCRESAHA.115.304262 PMC8760889 24903103

[44] Shemyakin A (2012). Arginase inhibition improves endothelial function in patients with coronary artery disease and type 2 diabetes mellitus. Circulation.

[45] Kovamees O, Shemyakin A, Pernow J (2016). Amino acid metabolism reflecting arginase activity is increased in patients with type 2 diabetes and associated with endothelial dysfunction. Diabetes Vasc. Dis. Res..

[46] Feron O, Balligand J-L (2006). Caveolins and the regulation of endothelial nitric oxide synthase in the heart. Cardiovasc. Res..

[47] Bist A, Fielding PE, Fielding CJ (1997). Two sterol regulatory element-like sequences mediate up-regulation of caveolin gene transcription in response to low density lipoprotein free cholesterol. Proc. Natl. Acad. Sci. USA.

[48] Feron O, Dessy C, Moniotte S, Desager JP, Balligand JL (1999). Hypercholesterolemia decreases nitric oxide production by promoting the interaction of caveolin and endothelial nitric oxide synthase. J. Clin. Invest..

[49] Mineo C, Shaul PW (2012). Regulation of eNOS in caveolae. Adv. Exp. Med. Biol..

[50] Feron O, Saldana F, Michel JB, Michel T (1998). The endothelial nitric-oxide synthase-caveolin regulatory cycle. J. Biol. Chem..

[51] McDonald KK, Zharikov S, Block ER, Kilberg MS (1997). A caveolar complex between the cationic amino acid transporter 1 and endothelial nitric-oxide synthase may explain the ‘arginine paradox’. J. Biol. Chem..

[52] Li C, Huang W, Harris MB, Goolsby JM, Venema RC (2005). Interaction of the endothelial nitric oxide synthase with the CAT-1 arginine transporter enhances NO release by a mechanism not involving arginine transport. Biochem. J..

[53] Goldstein JL, Brown MS (2015). A century of cholesterol and coronaries: from plaques to genes to statins. Cell.

[54] Silverman MG (2016). Association between Llowering LDL-C and cardiovascular risk reduction among different therapeutic interventions: a systematic review and meta-analysis. JAMA.

[55] Kratzer A, Giral H, Landmesser U (2014). High-density lipoproteins as modulators of endothelial cell functions: alterations in patients with coronary artery disease. Cardiovasc Res.

[56] Woollett LA (2011). Review: Transport of maternal cholesterol to the fetal circulation. Placenta.

[57] Furuhashi M (1989). Expression of low density lipoprotein receptor gene in human placenta during pregnancy. Mol Endocrinol.

[58] Wadsack C (2003). Selective cholesteryl ester uptake from high density lipoprotein by human first trimester and term villous trophoblast cells. Placenta.

[59] Stefulj J (2009). Human endothelial cells of the placental barrier efficiently deliver cholesterol to the fetal circulation via ABCA1 and ABCG1. Circ Res.

[60] Mineo C, Shaul PW (2012). Functions of scavenger receptor class B, type I in atherosclerosis. Curr Opin Lipidol..

