# Supplementary material for: Maternal supraphysiological hypercholesterolemia associates with endothelial dysfunction of the placental microvasculature
Source: Sci Rep. 2018 May 16;8:7690. doi: 10.1038/s41598-018-25985-6 (PMC5955926; doi:10.1038/s41598-018-25985-6)
Supplement: Supplementary file 1 — Supplementary information [file 41598_2018_25985_MOESM1_ESM.pdf]

Maternal supraphysiological hypercholesterolemia associates with endothelial dysfunction of the placental microvasculature

[1]Bárbara Fuenzalida, [1]Bastián Sobrevia, [1]Claudette Cantin, [1]Lorena Carvajal, [1]Rocío Salsoso, [1,2]Jaime Gutiérrez, [1]Susana Contreras-Duarte, [1,3,4] Luis Sobrevia, [1]*Andrea Leiva

[1]Cellular and Molecular Physiology Laboratory (CMPL), Division of Obstetrics and Gynaecology, School of Medicine, Faculty of Medicine, Pontificia Universidad Católica de Chile, Santiago 8330024, Chile.

[2] Cellular Signalling and Differentiation Laboratory (CSDL), School of Medical Technology, Health Sciences Faculty, Universidad San Sebastian, Santiago 7510157, Chile.

[3]Department of Physiology, Faculty of Pharmacy, Universidad de Sevilla, Seville E-41012, Spain.

[4]University of Queensland Centre for Clinical Research (UQCCR), Faculty of Medicine and Biomedical Sciences, University of Queensland, Herston, QLD 4029, Queensland, Australia.

***Correspondence**: Dr Andrea Leiva
Division of Obstetrics and Gynaecology
School of Medicine, Faculty of Medicine
Pontificia Universidad Católica de Chile
P.O. Box 114-D, Santiago 8330024, Chile.
Telephone: +562-23548116
E-mail: aaleiva@uc.cl

Representative figures included in the Manuscript (representative from blots 1to 5) .

Figure 2C.

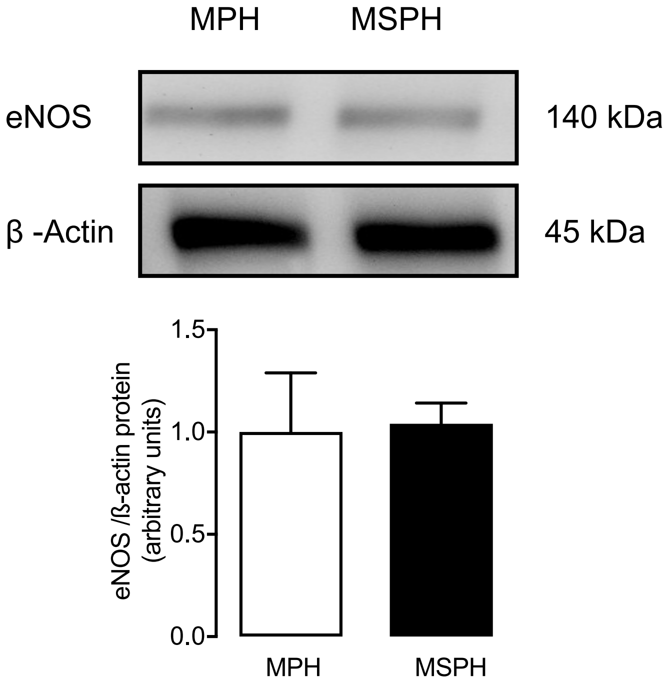

Figure 2. NOS activity. a). L-Citrulline formation from L-arginine in hPMEC from pregnancies in which the mother exhibited MPH or MSPH, in the absence (☐) or presence (■) of 100 µmol/L *N*^G^-nitro-L-arginine methyl ester (L-NAME, 30 minutes). **b).** L-Citrulline formation inhibited by L-NAME (from the data in **a**) in hPMEC from MPH and MSPH. **c).** Representative western blot for total eNOS (eNOS) in MPH or MSPH cells (ß-actin: internal control). *Lateral panel*: eNOS/ß-actin ratio densitometries normalized to 1 in MPH. Full-length blots are presented in Supplementary figures 1 and 2. In **(a)**, **P*<0.05 versus values in the absence of L-NAME, †*P*<0.05 versus MPH values. In **(b)**, **P*<0.05 versus MPH values. Values are mean ± S.E.M. (*n* = 6).

Representative figures included in the Manuscript (representative from blots 1to 5) .

Figure 3C.

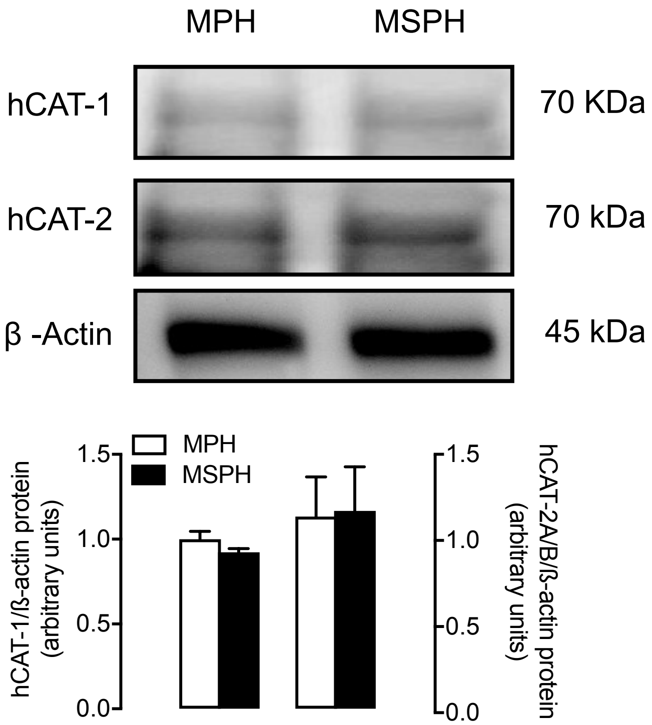

Figure 3. L-arginine transport. a). Uptake of 125 μmol/L L-arginine (1 minute, 37ºC) in hPMEC from pregnancies in which the mother exhibited MPH (□) or MSPH (■). **b).** Saturable L-arginine transport (0-1000μmol/L, 1 minute, 37ºC) in MPH or MSPH as in **a**. **c).** Representative western blots for hCAT-1 and hCAT-2A/B (hCAT-2) in hPMEC from MPH or MSPH (ß-actin: internal control). *Lateral panel*: hCAT-1 or hCAT-2/ß-actin ratio densitometries normalized to 1 in MPH. Full-length blots are presented in Supplementary figures 1 and 2 **P*<0.05 versus MPH values.Values are mean ± S.E.M. (*n* = 6).

Representative figures included in the Manuscript (representative from blots 1to 5) .

Figure 4B.

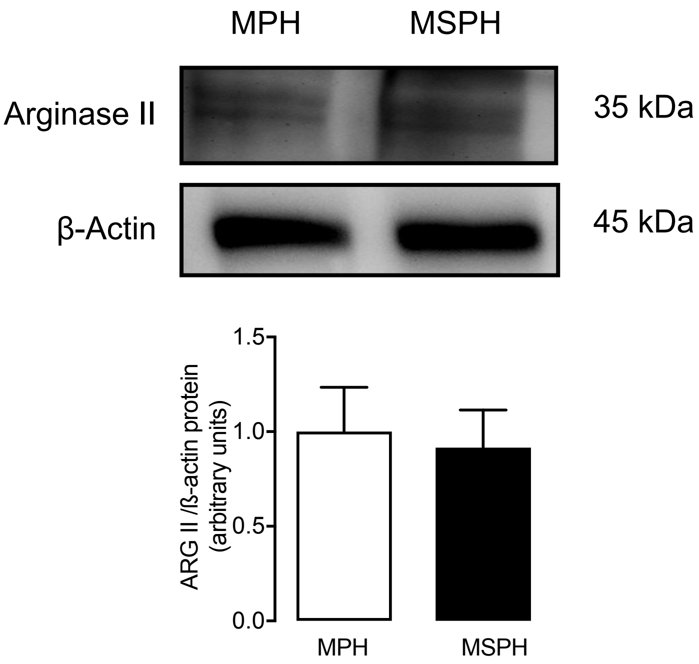

Figure 4. Arginases activity. a). Arginases activity evaluated as urea formation from L-arginine (50 mmol/L, 60 minutes, 37ºC) in hPMEC from pregnancies in which the mother exhibited MPH or MSH (see methods). **b).** Representative western blot for arginase II in hPMEC from MPH or MSPH (ß-actin: internal control). *Lateral panel*: arginase II/ß-actin ratio densitometries normalized to 1 in MPH. Full-length blots are presented in Supplementary figures 1 and 2 .*$P<0.05$ versus MPH values. Values are mean ± S.E.M. ($n = 6$).

Supplementary figure 1: western blot for human placental microvascular endothelial cells from MPH (samples MPH 1, 2, 3and 4) or MSPH (samples MSPH 1,2 and 3) placentas (see methods section). All the proteins were tested in the same membrane.

Western blot 1

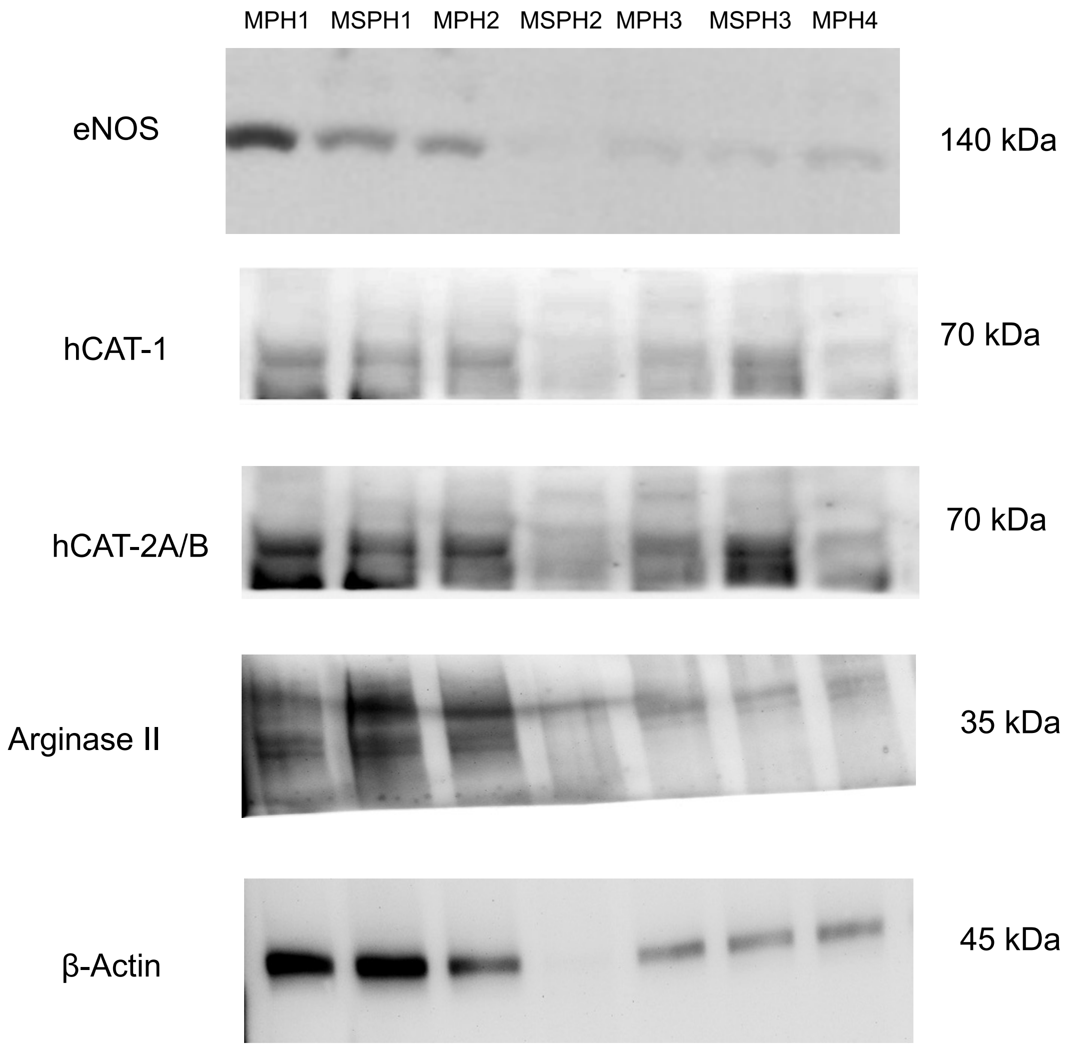

Supplementary figure 2: western blot for human placental microvascular endothelial cells from MPH (samples MPH 1, 2, 3, 5, 6 and 7) or MSPH (samples MSPH 4, 5, 6 and 7) placentas (see methods section). eNOS, Arginase II, hCAT-1 and 2 A/B were tested in the different membranes.

Western blot 2

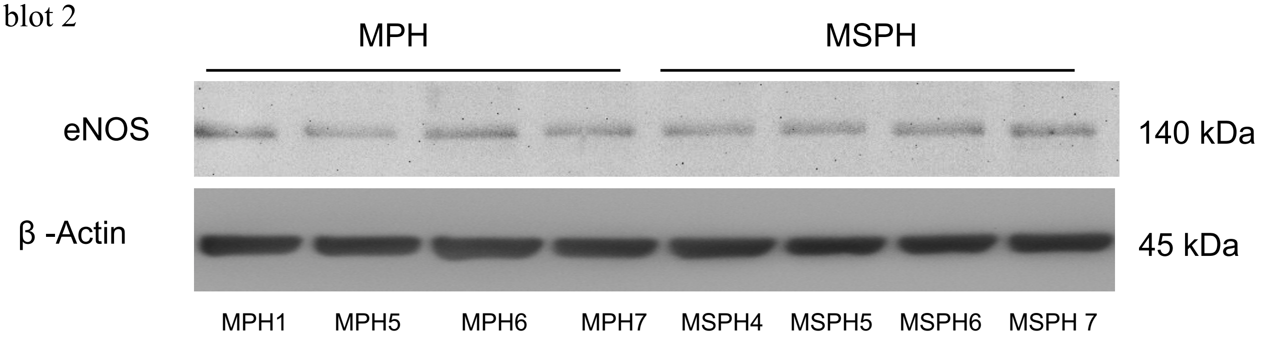

Western blot 3

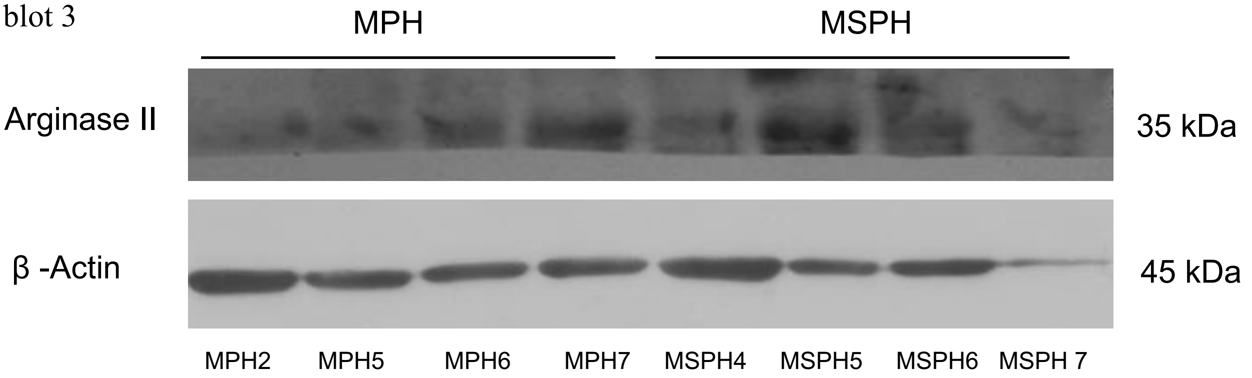

Western blot 4

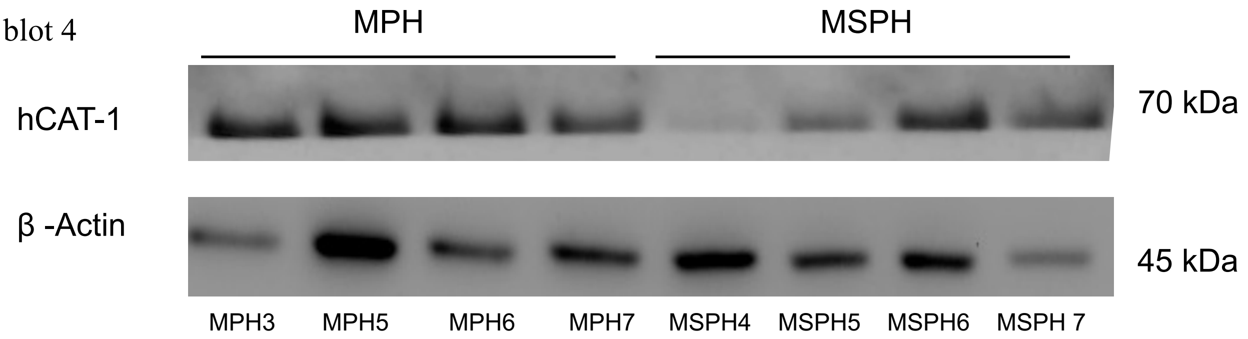

Western blot 5

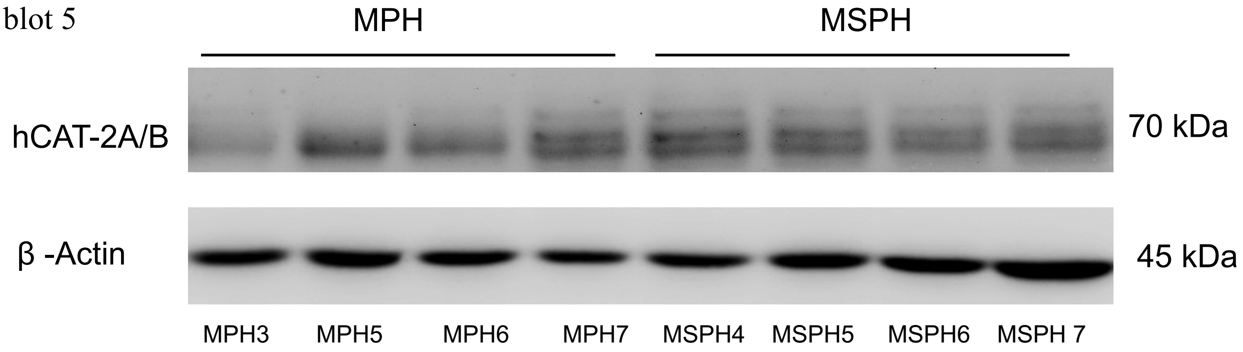

Supplementary data: Quantification of western blot 1to 5

BLOT 1										
Line	Sample	b-Actin	eNOS	eNOS/b-actin	Mean (MPH)	Relative units	hCAT-1	hCAT-1/b-actin	Mean (MPH)	Relative units
1	MPH1	11.655.974	12.402.368	1,06	1,06	1,00	2.358.560	0,20	0,40	0,51
3	MPH2	5.733.832	4.572.983	0,80		0,75	2.567.530	0,45		1,12
5	MPH3	2.957.811	1.531.770	0,52		0,49	1.748.196	0,59		1,48
7	MPH4	2.672.761	2.139.154	0,80		0,76	939.719	0,35		0,88
2	MSPH1	11.664.246	5.827.225	0,50		0,47	2.380.581	0,20		0,51
6	MSPH3	2.588.104	1.063.062	0,41		0,39	1.688.439	0,65		1,63
4	MSPH2	not determined								

BLOT 1										
Line	Sample	b-Actin	hCAT-2	hCAT-2/b-actin	Mean (MPH)	Relative units	Arg II	Arg II/b-actin	Mean (MPH)	Relative units
1	MPH1	11.655.974	3.126.116	0,27	0,40	0,67	1.776.527	0,15	0,19	0,80
3	MPH2	5.733.832	2.396.338	0,42		1,04	707.598	0,12		0,65
5	MPH3	2.957.811	1.668.246	0,56		1,41	680.654	0,23		1,21
7	MPH4	2.672.761	923.548	0,35		0,86	653.888	0,24		1,29
2	MSPH1	11.664.246	2.382.581	0,20		0,51	2.051.104	0,18		0,93
6	MSPH3	2.588.104	2.428.903	0,94		2,35	677.459	0,26		1,38
4	MSPH2	not determined								

BLOT 2						
Line	sample	b-Actin	eNOS	eNOS/b-actin	Mean (MPH)	Relative units
1	MPH1	6.882.841	6.858.205	1,00	0,73	1,36
2	MPH5	6.426.012	3.482.719	0,54		0,74
3	MPH6	6.799.255	4.960.083	0,73		1,00
4	MPH7	6.134.598	3.993.033	0,65		0,89
5	MSPH4	7.111.255	3.984.790	0,56		0,77
6	MSPH5	7.145.719	3.977.719	0,56		0,76
7	MSPH6	7.247.669	5.165.962	0,71		0,98
8	MSPH7	7.139.134	4.824.083	0,68		0,93

BLOT 3						
Line	sample	b-Actin	Arg II	Arg II/b-actin	Mean (MPH)	Relative units
1	MPH2	11.337.276	778.650	0,07	0,29	0,24
2	MPH5	7.908.790	1.037.548	0,13		0,45
3	MPH6	7.528.033	2.924.205	0,39		1,34
4	MPH7	7.587.619	4.298.225	0,57		1,95
5	MSPH4	11.178.983	1.335.891	0,12		0,41
6	MSPH5	6.401.205	1.805.912	0,28		0,97
7	MSPH6	7.469.962	1.678.523	0,22		0,77
8	MSPH7	1.755.527	869.577	0,50		1,71

BLOT 4						
Line	sample	b-Actin	hCAT-1	hCAT-1/b-actin	Mean (MPH)	Relative units
1	MPH3	1.785.083	9.095.338	5,10	3,00	1,70
2	MPH5	5.989.518	9.320.338	1,56		0,52
3	MPH6	2.598.326	8.110.631	3,12		1,04
4	MPH7	2.871.033	6.405.439	2,23		0,74
5	MSPH4	3.880.376	962.477	0,25		0,08
6	MSPH5	2.199.841	3.670.246	1,67		0,56
7	MSPH6	2.725.497	7.571.146	2,78		0,93
8	MSPH7	990.477	6.494.995	6,56		2,19

BLOT 5						
Lane	sample	b-Actin	hCAT-2	hCAT-2/b-actin	Mean (MPH)	Relative units
1	MPH3	6.526.154	7.765.016	1,19	1,68	0,71
2	MPH5	7.333.740	12.626.108	1,72		1,02
3	MPH6	6.816.569	10.958.865	1,61		0,96
4	MPH7	5.043.255	11.114.522	2,20		1,31
5	MSPH4	6.049.083	13.628.886	2,25		1,34
6	MSPH5	7.591.104	13.113.765	1,73		1,03
7	MSPH6	7.417.619	10.311.208	1,39		0,83
8	MSPH7	10.216.104	13.533.451	1,32		0,79
